# Establishment of an Undergraduate FOAM Initiative: International Emergency Medicine (iEM) Education Project for Medical Students

**DOI:** 10.5811/westjem.2020.10.48385

**Published:** 2020-12-16

**Authors:** Elif D. Cakal, Arif A. Cevik, Lit S. Quek, Abdel Noureldin, Fikri Abu-Zidan

**Affiliations:** * University of Dundee, School of Medicine, Centre for Medical Education, Dundee, United Kingdom; † United Arab Emirates University, College of Medicine and Health Sciences, Department of Internal Medicine, Al Ain, United Arab Emirates; ‡ National University of Singapore, School of Medicine, Department of Emergency Medicine, Singapore; § Tawam Hospital, Department of Emergency Medicine, Al Ain, United Arab Emirates; ¶ Pinckneyville Community Hospital, Department of Emergency Medicine, Pinckneyville, Illinois; || United Arab Emirates University, College of Medicine and Health Sciences, Department of Surgery, Al Ain, United Arab Emirates

## Abstract

**Introduction:**

Our goal was to describe the structure, process, platforms, and piloting period activities of the International Emergency Medicine (iEM) Education Project, which is a Free Open Access Medical Education (FOAM) initiative designed for medical students.

**Methods:**

This was a descriptive study. We analyzed the activity data of iEM Education Project platforms (website and image, video, audio archives) in the piloting period (June 1, 2018–August 31, 2018). Studied variables included the total and monthly views, views by country and continents, the official languages of the countries where platforms were played, and their income levels.

**Results:**

Platforms were viewed or played 38,517 times by users from 123 countries. The total views and plays were 8,185, 11,896, and 18,436 in June, July, and August, respectively. We observed a monthly increasing trend in all platforms. Image archive and website were viewed the most. All platforms were dominantly viewed from Asia and North America, high- and upper-middle-income countries, and non-English speaking countries. However, there were no statistically significant differences between continents, income levels, or language in platforms, except for the website, the project’s main hub, which showed a strong trend for difference between income levels (Kruskal-Wallis, P = 0.05). Website views were higher in high-income countries compared with low- and lower-middle income countries (Mann Whitney U test, P = 0.038 and P = 0.021, respectively).

**Conclusion:**

The iEM Education Project was successfully established. Our encouraging initial results support the international expansion and increased collaboration of this project. Despite targeting developing countries with limited resources in this project, their engagement was suboptimal. Solutions to reach medical students in these countries should be investigated.

## INTRODUCTION

Methods of learning medicine have changed dramatically over the last decade. Web-based learning has revolutionized medical education by allowing information to be shared rapidly without borders, supporting individual needs.^1^, ^2^ Advancements in web technology encourage physicians to participate globally and benefit from collective intelligence.^3^ After the term “Free Open Access Medical Education (FOAM)” was established, online medical learning gained fresh momentum, and a vibrant digital community was born.^4^ Finally, through social media, interactions within this global community reached its peak.^5^ The Emergency Medicine and Critical Care (EMCC) community, in particular, has been the leader of the FOAM movement.^6^–^8^

The FOAM resources targeting undergraduate medical education is lagging behind those targeting postgraduate education.^9^,^10^ This lag may be attributed to the topics favored by FOAM.^10^ Research and technical aspects, which appeal more to postgraduate students, are over-represented compared with core concepts that are of interest to medical students.^9^, ^10^ Increasing FOAM resources covering fundamental concepts may encourage medical students to engage with FOAM. FOAM is a valuable learning tool in undergraduate medical education that fits modern educational methods and instructional strategy.^11^ It serves as a complement or an alternative to the traditional instructional techniques.^12^–^14^ Medical students appreciate FOAM because of its effectiveness, time-efficiency, convenience, and fun.^12^, ^13^, ^15^, ^16^ It can also save money and instructor time, compared with traditional techniques.^11^

FOAM may also benefit undergraduate training in low- and middle-income countries (LMICs). In LMICs, staffing emergency departments (ED) is so big a challenge that medical graduates without further residency training work as independent physicians in EDs in some settings.^17^ To overcome this challenge, more healthcare staff must be trained; however, the scarcity of medical professionals hinders the education of future generations, thereby causing a vicious cycle.^18^ Educating local physicians in collaboration with the international emergency medicine (EM) community was suggested as a potential solution to break this cycle.^19^ Electronic learning, particularly FOAM can facilitate international collaboration by removing geographical boundaries, and reduce the dependence on local educators by giving access to otherwise expensive information, saving trainers’ time for hands-on training, and making information available at the point of care.^11^, ^19^–^22^

The International Emergency Medicine (iEM) Education Project is a new international undergraduate FOAM initiative, which is aligned with the international undergraduate EM curricula recommendations.^23^, ^24^ This project aims to promote and support undergraduate EM training internationally, especially in LMICs, and provide free learning resources for medical students and educators. It has five platforms: a website as the main hub;^25^ image archive;^26^ video archive;^27^ audio archive;^28^ and social media. We aim to describe the structure, process, and piloting period activities of the iEM Education Project.

## METHODS

### Ethical Considerations

According to the United Arab Emirates University (UAEU) Research and Graduate Studies Ethics Guidelines, this study did not require an ethical approval process (an exemption) because it did not include human subjects and identifiers.

### Structure and Process of the Project

**Figure 1:**
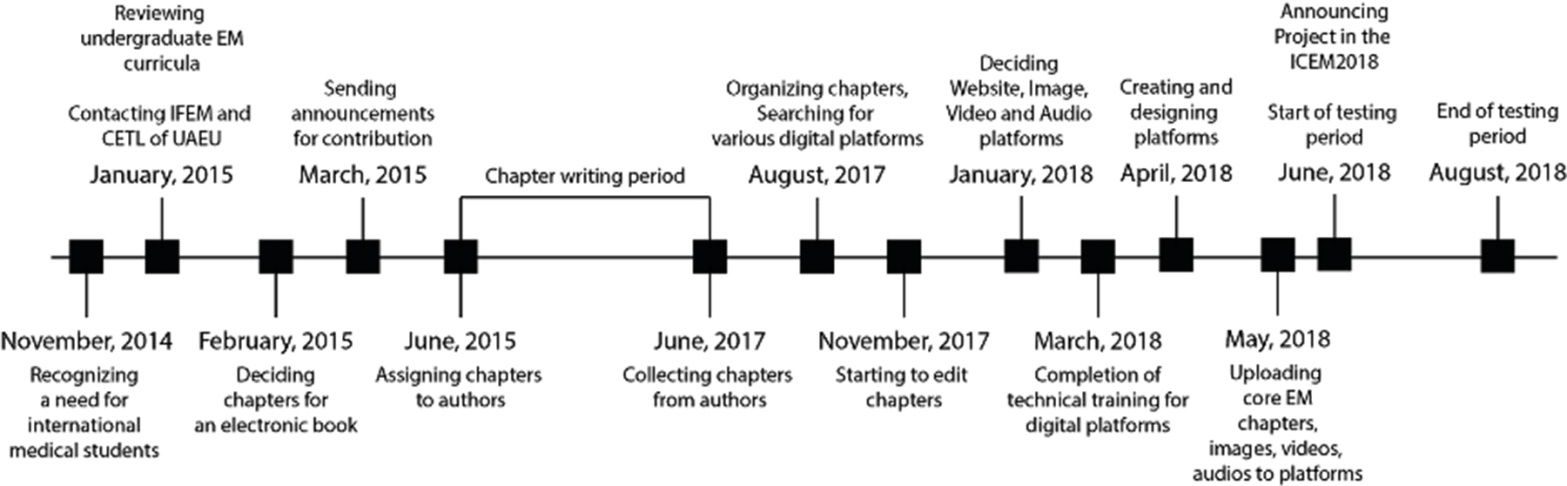
Timeline for constructing the International Emergency Medicine Education Project. *EM*, emergency medicine; *IFEM*, International Federation for Emergency Medicine; *CETL*, Center for Excellence in Teaching and Learning; *UAEU*, United Arab Emirates University; *ICEM*, International Conference on Emergency Medicine.

Upon acknowledging the need for EM resources for medical students, three academic scholars (AAC, LSQ, AN) decided to create an electronic textbook for international medical students and interns at the end of 2014, laying the foundations of the iEM Education Project (Figure 1). English was chosen as the language of the project because it is the most prevalent language in the world.^29^ One hundred six chapter titles were determined in alignment with the International Federation for Emergency Medicine (IFEM) and Society for Academic Emergency Medicine undergraduate curricula recommendations.^23^,^24^ Chapters’ internal structure was aligned with the natural flow of patient-physician interaction and mainly focused on the critical actions of the EM. To find authors, an email containing the selected chapter titles and asking for volunteers’ interest areas were distributed through the email groups of the IFEM, American College of Emergency Physicians International (ACEP-Int), and Council of Emergency Medicine Residency Directors (CORD-EM) in the first quarter of 2015. Answering the call were 133 contributors from 19 countries. In mid-2015, the chapters were assigned to the contributors according to their preference when possible, and most chapters were gathered until mid-2017. From the last quarter of 2017 to the middle of 2018, chapters written by contributors were reviewed by four editors in terms of content and format. Additionally, chapters were reviewed by a native English-language editor. Such generous support encouraged the founders to expand the project. After six months of online training and the development of infrastructure, a website including book chapters and blog, clinical and radiological image archive, video archive, audio archive, and social media accounts were initiated in May 2018.^25^ The iEM Education Project was first officially announced at the IFEM’s International Conference on Emergency Medicine in Mexico City, Mexico, in June 2018, and its platforms were advertised regularly through social media.

The book chapters and blogposts were shared via a website placed in WordPress (Automattic Inc., San Francisco, CA), an open-source content management system.^30^ The images were placed in the photo management and sharing application, Flickr (Mountain View, CA).^31^ Videos that are used in blogposts and book chapters were placed on YouTube (San Bruno, CA), a free video-sharing platform.^32^ Book chapters were voiced over digitally by the Amazon Polly application^33^ and placed in the open audio platform, SoundCloud (Berlin, Germany).^34^ All iEM Education Project contents were published with the project logo, content providers’ names, and were licensed under a Creative Commons Attribution-NonCommercial-ShareAlike 4.0 international license.^35^

Creating such a project required considerable support from various stakeholders. In addition to IFEM, which endorsed the project from the beginning, several international EM associations, including ACEP-Int and CORD-EM supported and promoted the project during the preparatory phase and piloting period. Through associations’ networks, we were able to gain support from the international EM community and recruit authors. UAEU supported the project financially. To keep expenses low, editors avoided professional services and processed most technical tasks themselves, including editing chapters, creating and designing platforms, creating and editing visual, audio and video files, and uploading all materials to relevant platforms. One of the editors received a total of approximately 22 hours of online training on electronic learning, WordPress, and search engine optimization. Additionally, depending on the length and content of the chapter, each chapter necessitated 10–20 hours of editors’ work. The initial expenses were around 1,500 United States dollars, including costs of website hosting services, plugins, graphic design platforms, and applications.

### Studied Variables

This is a descriptive study. We analyzed the data of the piloting period. During this piloting period, we aimed to explore the usage of project platforms and included three months from the announcement date (from June 1, 2018–August 31, 2018). During this period, the project had five active channels, namely, website, image, video, audio archives, and social media. WordPress, Flickr, YouTube, SoundCloud, and Twitter (San Francisco, CA) were the hosting platforms of these channels, respectively. Each hosting platform provides anonymous activity data to the administrator. Only data from the hosting platforms were used in the analysis. We did not include social media activity in the analysis. Studied variables included the total and monthly views, views by country and continents, the official language of the countries, and their income level according to World Bank data for 2018 (low, lower-middle, upper-middle, high).

### Statistical Analysis

We extracted available anonymous data to Microsoft Excel sheets (Microsoft Corporation, Redmond, WA). The data was coded and cleaned for statistical analysis. The Shapiro-Wilk test was used for normal distribution analysis of the data. We used non-parametric tests because the distribution of the data was not normal. Categorical data are presented as frequency (%), and continuous variables are presented as median (range), where appropriate. Continuous variables were assessed with the Mann-Whitney U test (for two independent groups) and Kruskal-Wallis test (for more than two independent groups). The significance level was determined at 0.05. Statistical analyses were performed using the Statistical Package for the Social Sciences version 26 (IBM Corp, Armonk, NY).

## RESULTS

In total, the iEM Education Project platforms were viewed or played 38,517 times by users from 123 countries. The total views and plays were 8,185, 11,896, 18,436 in June, July, and August, respectively (Table 1).

### Website

**Figure 2:**
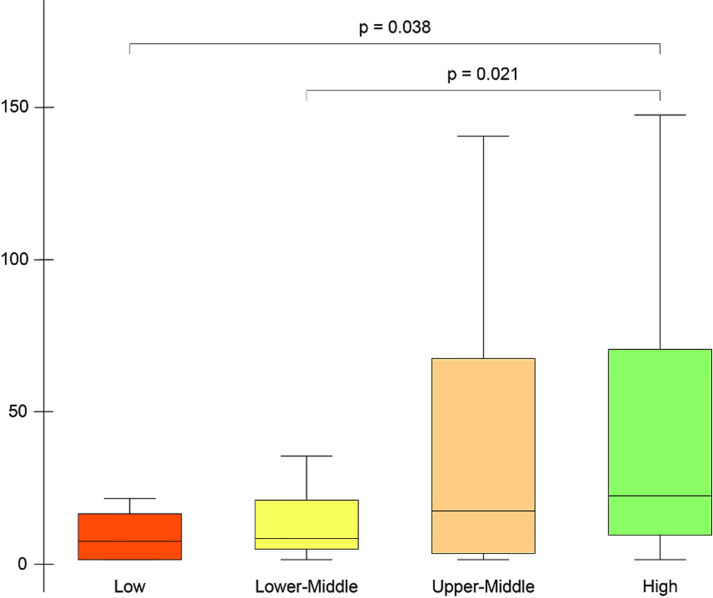
Box-and-whiskers comparison plot of website views by income levels. Each box shows the Interquartile range (from the 25th–75th percentile). The horizontal line within each box represents the median. P-values by Kruskal-Wallis tests. Outliers have been removed from the figure.

The website is the main hub of the project. At the end of the piloting period, 85 chapters and 126 blogposts were published on the website and viewed a total of 13,778 times by users from 117 countries. The number of views was 2,484, 4,444, and 6,850 in June, July, and August, respectively. Of those views, 76.9% were from Asia and North America and 94.4% of views were from high- and upper-middle-income countries, while 5.6% were from LMICs. Views from countries of which the official language was not English constituted 74% of the total. The views according to continents, income levels, and languages are given in Table 2. There was no difference in views between continents (Kruskal-Wallis, *P* = 0.244), and language (Mann Whitney U test, *P* = 0.865). There was a strong trend for difference between income levels by views (Kruskal-Wallis, *P* = 0.05), the views were higher in high-income countries compared with low- and lower-middle-income countries (Mann Whitney U test, *P* = 0.038 and *P* = 0.021, respectively) (Figure 2).

### Image Archive

Until the end of the piloting period, 674 visuals were published on Flickr image archive and viewed a total of 23,129 times. The number of views was 5391, 7035, and 10,703 in June, July, and August, respectively.

### Video Archive

Until the end of the piloting period, 107 videos were published on YouTube video archive and viewed a total of 1,176 times by users in 60 countries. The number of views was 237, 290, and 649 in June, July, and August, respectively. Asia (54.3%), North America (29.2%), and Europe (9.2%) were the top three continents where the video content was viewed. High- and upper-middle-income countries constituted 90.2% of the views. Non-English-speaking countries accounted for 74.7% of the views. There were no differences in views by continents (Kruskal-Wallis, *P* = 0.350), income levels (Kruskal-Wallis, *P* = 0.627), and language (Mann-Whitney U test, *P* = 0.840).

### Audio Archive

Until the end of the piloting period, 41 audio chapters were published on SoundCloud audio archive and played a total of 434 times by users in 41 countries. The number of views was 73, 127, and 234 in June, July, and August, respectively. Asia (39.2%) and North America (32.5%), and Europe (13.4%) were the top three continents where users played the audio content. High- and upper-middle-income countries constituted 91.7% of the plays. Non-English-speaking countries accounted for 61.9% of the plays. There were no differences in plays by continents (Kruskal-Wallis, *P* = 0.349), income levels (Kruskal-Wallis, *P* = 0.309), and language (Mann-Whitney U test, *P* = 0.508).

## DISCUSSION

In this study, we analyzed the piloting period data of the iEM Education Project, an EM FOAM project devoted to undergraduate EM training. The platforms were ranked in decreasing order of views as Flickr image archive, website, YouTube video archive, and SoundCloud audio archive. The monthly increase in views and plays in the piloting period encouraged us to continue publishing and expanding the project. The majority of views and plays were from high and upper-middle-income countries. Although all channels were in English, countries where English is not the official language used the source more often.

Little has been published about the activity data of FOAM projects, much less regarding their early periods. Acilci.net, a FOAM blog in Turkish, was reported to have been visited 3,500 times in its first month.^36^ St.Emlyn’s blog grew from 3497 to 36,377 average monthly views between 2012–2016.^37^ Other reports from the Academic Life in Emergency Medicine blog examined the activity and impact of FOAM for specific topics such as resident well-being, team-based learning, and resident teachers.^38^–^40^ All three reports demonstrated the potential of FOAM to be an asynchronous learning tool and “global classroom.” The iEM Education Project book chapters and blog were viewed over 13,500 times in the piloting period. All platforms included, over 38,500 views and plays from 123 countries were recorded. Among them, website and image archive were viewed the most. While the different amounts of content in each platform make the comparison of usage difficult, the relatively high traffic on website and image archive made us put more effort into these platforms. Overall, the monthly increase in the use of all platforms encouraged us to continue to project with all of them.

The EMCC community has shown a growing interest in FOAM from the start, but EMCC resources designed for medical students remain limited so far.^4^, ^7^ Over 180 websites that publish EMCC-related topics in English existed in 2013.^7^ Despite this considerable number, EMCC FOAM resources were mostly directed at postgraduate training, probably too advanced for undergraduate training and unable to cover the entire core topics.^9^, ^10^, ^41^ Few websites explicitly focus on core concepts that medical students need.^9^, ^42^–^44^ The iEM Education Project is among the few FOAM resources that focuses on undergraduate EM training and follows international undergraduate curricula recommendations.

The majority of medical students seem to use online resources.^45^–^47^ In terms of the frequency of use or perceived usefulness, online resources ranked in the top three by medical students.^47^–^51^ Notably, Al-Hazmi reported that medical students used online resources more than textbooks.^49^ Despite the popularity of online resources among medical students, resources devoted to medical students remain limited. The considerable and increasing amount of activity in the piloting period indicates that the iEM Education Project contributed as a useful online resource for medical students.

Despite the widespread use of clinical images in undergraduate medical education, sourcing clinical images poses challenges both to medical students and educators. Medical images facilitate learning both core and complex concepts and improve memory.^52^ A recent survey demonstrated that 87% of medical students use clinical images.^53^ The Internet was the primary source (96%), and approximately one-third of medical students found it difficult to find appropriate images. The cost, validity, consent level, file size, and copyright status were important factors affecting students’ choice for using clinical images.^53^ Medical educators sourced medical images mainly from open-access search engines, social media, and student textbooks. Nevertheless, they were worried about copyright violation and consent level.^52^ In this regard, iEM Education Project Image Archive seems to address medical students’ and educators’ concerns by providing cost- and copyright-free medical images. This may explain why it is the most viewed iEM Education Project platform.

iEM Education Project platforms were accessed from five continents. In total, most views were from Asia and North America. Although views in the third month increased in all continents compared to their views in the first month, views from North America and South America showed a more prominent and regular increase. Various factors that might be affecting this distribution were suggested in the literature.^6^ Language seems to be one of the determinants.^6^ No previous study has extensively examined how language impacts access to FOAM, but few studies present controversial results. Overall, FOAM users are concentrated in English-speaking countries.^6^ However, 74% of views of the iEM Education Project website were from countries where English is not the official language. Interestingly, a FOAM blog publishing in Turkish reported that 10.6% of views were from 111 different countries, possibly doctors from Turkic origins.^36^ Similarly, the geographically dispersed access to iEM platforms may be attributed to the users who speak English as a primary or secondary language; however, built-in automatic translation placed on our website may also have facilitated the use of the website for users with little or no English proficiency.^36^ Our findings may imply that FOAM platforms in English seem to have considerable international reach, but how publishing in other prevalent or multiple languages might affect the international reach and the distribution of views remains to be discovered in future studies.

Another determinant of view distribution may be economics.^6^ A previous study, which reviewed approximately 18.7 million views of 12 FOAM blogs, found that views from high-income countries constituted approximately three-quarters of the total.^6^ Similarly, the iEM Education Project platforms were used dominantly from high-income and upper-middle-income countries. Even if the views from LMICs increased slightly each month, they constituted less than 6% of total views. Online learning was heralded as a feasible solution to challenges of medical education in resource-limited contexts, but in actuality the dissemination of FOAM remains limited in LMICs.^54^–^57^ The previous reports demonstrated that the impact of FOAM on LMICs was below expectations for several reasons. Infrastructure-related problems – such as limited access to computers and the Internet, and slow Internet speed; system-related problems such as the lack of systemic, curriculum-based approach; and general lack of awareness of FOAM, information and communications technology skills – hinder FOAM’s dissemination in LMICs.^54^–^57^ The low engagement from LMICs with the iEM Education Project may be a reflection of the constraints mentioned above and our marketing strategy.^6^ We advertised our project through international associations’ email groups and social media, in which the representation of LMICs might be less than ideal. Targeting more local EM organizations in LMICs could promote the project in areas in need more effectively. Nevertheless, more studies are needed to discover factors affecting the usage of online resources from different countries and regions.

## LIMITATIONS

We have to acknowledge that there are several limitations to this study. One major limitation is that although we targeted medical students and advertised our project accordingly, we could not confirm whether all viewers were medical students as targeted. At the start of the project, we refrained from requiring any kind of registration as it could have caused privacy concerns and discouraged users from using our platforms.^58^ Alternatives to registration include single sign-on. However, even these techniques do not guarantee that the provided personal information is correct.^58^ Our initial evaluation included a short period of time. Although an extended period could have revealed more information and strengthened the statistical analysis, we intentionally examined the activity data from the piloting period to address potential issues early. We have used data provided by hosting services, which we did not have control over. Furthermore, the lack of data about some platforms (eg, Flickr) limited our analysis. Using additional services (eg, Google Analytics) may have increased the types of data available. Finally, during the piloting period, the major means for promotion was through social media. The editors’ social network may have affected the countries that use iEM Education Project resources in the piloting period.

## CONCLUSION

The International Emergency Medicine (iEM) Education Project, a free open-access medical education resource devoted to medical students, was successfully established. Our encouraging initial results support the international expansion of and increased collaboration on this project. Despite targeting developing countries with limited resources, we found that their engagement was suboptimal. Solutions to reach medical students in these countries should be investigated more. We hope that reporting our experience and data may inspire educators to create more undergraduate FOAM resources and prepare them for upcoming challenges.

## Figures and Tables

**Table 1:** Views and plays according to platform.

Platform	Content	Views and plays N (%)				Number of countries

		June	July	August	Total	
Website	85 chapters and 126 blogposts	2,484 (30.4)	4,444 (37.4)	6,850 (37.2)	13,778 (35.8)	117
Image archive	674 visuals	5,391 (65.9)	7,035 (59.1)	10,703 (58.1)	23,129 (60.1)	N/A
Video archive	107 videos	237 (2.9)	290 (2.4)	649 (3.5)	1176 (3.1)	60
Audio archive	41 audio chapters	73 (0.9)	127 (1.1)	234 (1.3)	434 (1.1)	41
Total	1,033	8,185	1,1896	18,436	38,517	123*

* After excluding overlapping countries

N/A: Data was not available.

**Table 2:** The website views according to continents, income levels and language.

	Countries N (%) 117 (100)	Views N (%)			Total views N (%) 13,778 (100)	P-value

		June 2,484 (100)	July 4,444 (100)	August 6,850 (100)		
				
Continents						0.244
Africa	15 (12.8)	4 (0.2)	176 (3.9)	53 (0.8)	233 (1.7)	
Asia	37 (31.6)	1,391 (55.9)	2,204 (49.6)	1,855 (27.1)	5,450 (39.6)	
Europe	38 (32.5)	239 (9.6)	836 (18.8)	759 (11.1)	1,834 (13.3)	
North America	13 (11.1)	690 (27.8)	993 (22.3)	3,453 (50.4)	5,136 (37.3)	
Oceania/Australia	3 (2.6)	33 (1.3)	51 (1.1)	77 (1.1)	161 (1.2)	
South America	11 (9.4)	127 (5.1)	184 (4.1)	653 (9.5)	964 (6.9)	
Income level						0.05
Low	6 (5.2)	2 (0.08)	49 (1.1)	2 (0.03)	53 (0.4)	
Lower-middle	24 (20.5)	138 (5.6)	205 (4.6)	371 (5.4)	714 (5.2)	
Upper-middle	33 (28.2)	1,149 (46.3)	2,293 (51.6)	3,688 (53.8)	7,130 (51.7)	
High	54 (46.1)	1,195 (48.1)	1,897 (42.7)	2,789 (40.7)	5,881 (42.7)	
Language						0.865
English	28 (23.9)	598 (24.1)	1,113 (25.1)	1880 (27.4)	3,591 (26.0)	
Non-English	89 (76.1)	1,886 (75.9)	3,331 (74.9)	4,970 (72.6)	10,187 (74.0)	
